# A Federated Blockchain Architecture for File Storage with Improved Latency and Reliability in IoT DApp Services

**DOI:** 10.3390/s23208569

**Published:** 2023-10-18

**Authors:** Dongjun Na, Jinbum Kim, Juseong Jeon, Sejin Park

**Affiliations:** Department of Computer Engineering, Keimyung University, Daegu 42601, Republic of Korea; kmu5544616@gmail.com (D.N.); jinbum9958@gmail.com (J.K.); mo66jyp@gmail.com (J.J.)

**Keywords:** internet of things, Web 3.0, Ethereum, application platform, service-oriented architecture, blockchain-based storage

## Abstract

Blockchain technology can address data falsification, single point of failure (SPOF), and DDoS attacks on centralized services. By utilizing IoT devices as blockchain nodes, it is possible to solve the problem that it is difficult to ensure the integrity of data generated by using current IoT devices. However, as the amount of data generated by IoT devices increases, scalability issues are inevitable. As a result, large amounts of data are managed on external cloud storage or distributed file storage. However, this has the disadvantage of being outside the blockchain network. This makes it difficult to ensure reliability and causes high latency during data download and upload. To address these limitations, we propose a method for managing large amounts of data in the local storage node of a blockchain network with improved latency and reliability. Each blockchain network node stores data, which is synchronized and recovered based on reaching a consensus between smart contracts in a cluster network. The cluster network consists of a service leader node that serves as a gateway for services and a cluster node that stores service data in storage. The blockchain network stores synchronization and recovery metadata created in the cluster network. In addition, we showed that the performance of smart contract execution, network transmission, and metadata generation, which are elements of the proposed consensus process, is not significantly affected. In addition, we built a service leader node and a cluster node by implementing the proposed structure. We compared the performance (latency) of IoT devices when they utilized the proposed architecture and existing external distributed storage. Our results show improvements up to 4 and 10 times reduction in data upload (store) and download latency, respectively.

## 1. Introduction

Blockchain technology was first developed to enable distributed smart contracts, and its popularity has grown owing to cryptocurrency networks such as Bitcoin [1]. Later, blockchain platforms supporting smart contracts such as Ethereum [2] and Hyperledger Fabric [3] emerged, making it possible to process multi-purpose transactions. With smart contracts, blockchains manage distributed nodes executing intelligent logic in peer-to-peer (P2P) [4] networks rather than a central trustee; this ensures the reliability of logic and data storage. Because of these advantages, recent generations of decentralized networks, namely Web 3.0 [5,6], rely on blockchain technology. Web 3.0 orchestrates data through a decentralized blockchain network. More specifically, its trusted services, enabled by smart contracts, are transmitted, processed, and stored in a blockchain network such as Ethereum rather than a central server. In addition, decentralized nodes provide services less affected by attacks such as denial of service (DoS) and data tampering by central administrators, thereby reducing hacking and data leakage risks. According to Gartner [7], 63% of digital marketing leaders struggle to deliver personalized experiences, and approximately 84% of digital marketers use artificial intelligence and machine learning, the technology driving Web 3.0 for real-time personalization. These together improve marketing capabilities that deliver a better experience. In addition to Web 2.0, which is currently in use on the Internet, Web 3.0 is a distributed network organized around user data and permissions and implemented using blockchain technology and smart contracts. Similarly, IoT devices can leverage the blockchain technology that Web 3.0 uses to achieve the same positive effects that Web 2.0 has over Web 3.0. IoT devices can collect and process data from the physical world through sensors and actuators. The data they collect can be executed with smart contracts, using distributed ledger technologies like blockchain to ensure immutability and security. Additionally, IoT devices can also be used to securely manage and control the permissions and data that they produce. For example, IoT devices can use blockchain technology to secure user privacy and collect or control data with user consent through smart contracts. Therefore, IoT devices can become more powerful when used in conjunction with blockchain and smart contracts, but are limited by the low performance caused by the blockchain’s interaction with external storage, such as the following.

### 1.1. Motivation

The data generated by a blockchain network built with IoT devices is stored equally across all IoT devices participating in the blockchain network. As a result, the capacity requirements inside the network increase, which leads to the limitations of blockchain technology.

Therefore, Web 3.0 services with a network backend similar to blockchain networks implemented with IoT devices will also have large file data based on data storage built outside the blockchain network due to scalability issues.

According to Cloudwards.net [8], 94% of businesses use enterprise cloud services. Web 3.0 services [9,10,11] store data outside the blockchain based on the existing cloud and record only metadata in the blockchain. However, the cloud service-based data storage method cannot guarantee transparency and reliability due to its centralized structure, and file loss is possible. To address this limitation, most Web 3.0 services [12,13] use distributed storage network services [14,15] to store data.

**Figure 1:**
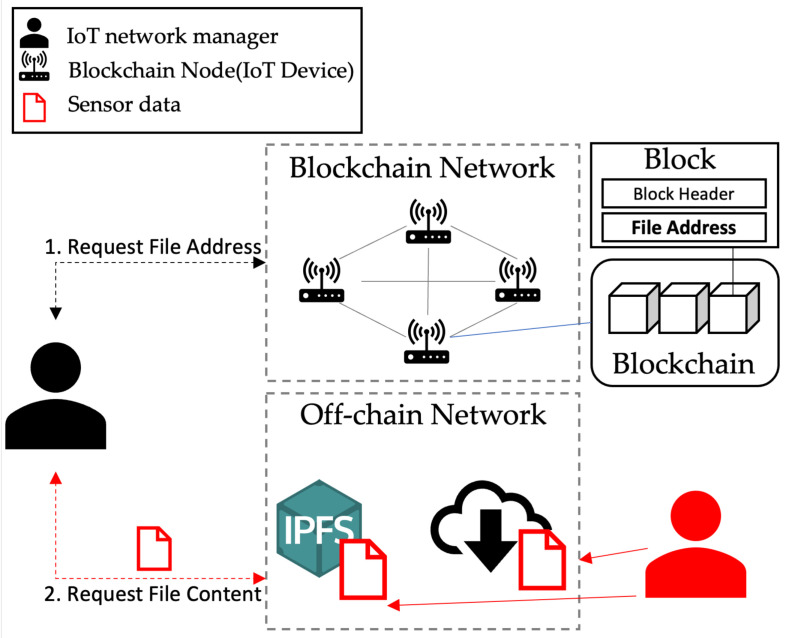
Data storage process of existing blockchain based services.

Figure 1 shows the data storage process in the existing Web 3.0. In this structure, the file-storing infrastructure and the blockchain network are separated. The client stores data in an external distributed file system and records metadata resulting from the data storage on the blockchain.

### 1.2. Challenges

In existing distributed storage networks, it is challenging to prevent file loss by distributing and storing data in only a part of the network owing to scalability problems. In addition, the increase in gateway traffic utilizing a centralized gateway for efficiency in a distributed environment can degrade performance and reliability. To address these limitations, the following challenges must be overcome:

- Difficult to prevent data loss: In the case of distributed storage configured externally by the blockchain, the node that maintains the file lacks the logic to store it, so it cannot prevent data from being lost.
- Possibility of data forgery: When file data are stored in a centralized structure such as cloud storage, there is a possibility of data forgery by an administrator.
- Centralization issues with gateways: In the case of existing distributed data storage, there is a gateway to efficiently process users’ data storage and download requests, but this causes centralization problems.
- Latency issues with I/O requests: High latency when requesting to download data stored in a distributed file system or external cloud storage must be addressed. In particular, when loading distributed files from a distributed system, there is a large amount of latency in the process of merging the system itself and the external download or upload over the Internet.

To address each of the challenges presented, we have the following contributions.

### 1.3. Contribution

The main contributions of this paper are listed as follows:

- File management method based on the service provider to prevent file loss. We propose a service-providing architecture for creating services in a blockchain network based on smart contracts to prevent file loss and a method for storing data in nodes participating in service creation and maintenance.
- Data consensus technique to ensure the integrity of user data based on original file replication and metadata. When storing user service data, the data of the distributed service nodes are synchronized by executing a synchronization agreement on the service data, and the reliability of the data queried by the client is ensured by performing recovery on the blockchain through metadata recorded on the blockchain. By applying this technique, the blockchain networks running on IoT devices can process data with high performance by requesting data from the blockchain network instead of the external storage network. Metadata are recorded on the blockchain to ensure reliability, and as data are managed locally rather than in an external network, this increases data processing latency while increasing performance.
- Distributed storage supporting high-performance data I/O based on blockchain network. To ensure the reliability of stored data, a technique is applied in which nodes in the blockchain network maintain service data.

To solve the storage limitation, a service cluster that provides storage resources for services in the blockchain network is formed. In a blockchain network, clusters can be built from multiple service units. In a blockchain network, clusters can be built from multiple service units. Therefore, cluster nodes will only retain data generated by the services to which they belong. Each service cluster also has a service leader node that acts as a gateway. The service leader node serves as a router that forwards data storage and query requests from clients who use the service through the participation of multiple nodes to the cluster node and is periodically replaced by applying a replacement policy to solve the centralization problem.

## 2. Related Work

In this section, we present existing studies for blockchain data storage and describe the main differences from our proposed architecture. Filecoin [16] operates on the upper layer of the interplanetary file system (IPFS) [17], and P2P-connected nodes store distributed files. A client can store files in a storage space with a storage provider. By consensus, there are Proof of Spacetime and Proof of Replication processes. Proof of Spacetime guarantees that the client stores files for a certain period, and Proof-of-Replication can guarantee that data are stored in a physical storage space. Swarm [18] is a basic layer service of Ethereum as a distributed storage platform and content distribution service. Swarm provides DApp developers with a foundation in messaging, data streaming, P2P statistics, variable resource update, storage provision, storage verification and proof of custody scan and repair, payment channels, and database service areas. In addition to the P2P storage function, Swarm is an incentive system that supports resource trading used in P2P. It provides a solution that implements DDoS attack prevention, zero-downtime, fault-tolerant, censorship-resistant, and self-sustaining functions. In Web3Storage [19], data are stored based on IPFS. IPFS nodes temporarily store data on three geographically distributed nodes and store that data on at least five distributed miners on the Filecoin network. This technique provides a simple interface through IPFS, and there is a node that hosts itself. Consequently, the service efficiency is high, but unlike the approach we propose, the solution for the delay between the user, the cluster, and the Filecoin network is not considered. In STORJ [20], a sharding technique is applied for data storage. The metadata of the sharded data are stored in the Ethereum blockchain, and the metadata includes location information from which the data can be retrieved. The shared data are merged into the client’s local system, and data forgery is periodically verified using the parity shard technique. Since this technique divides and encrypts files and stores them, there is a latency, and since the files are merged in the user’s local system, there is a load on the client. In BigChainDB [21], nodes in the network maintain MongoDB [22] to store data and Tendermint [23] for data consensus. By consensus based on Tendermint, up to 1/3 of Byzantium can be allowed, and stability exists by managing the list of nodes that the network subject will participate in. However, in the case of MongoDB, there is a limit to the amount of data that can be stored, and the network list is managed privately. In BlockHouse [24], all operations for data storage are performed through smart contracts based on a private blockchain. In addition, by applying the Proof of Retrievability System, data verification is performed over a set period. Once the data are verified, it is securely stored using a smart contract and encryption algorithm. Through this process, trust in the data stored on the server can be guaranteed based on the blockchain, but there is a limit to increasing the size of the blockchain. In our study, when a user makes a verification request, there is a difference as the metadata are managed on the public blockchain. In Pise et al. [25], only the hash value is recorded in the blockchain after encrypting and distributing the file for data storage. In our study, we propose blockchain-based cloud storage with data encryption as a safe and efficient way to store data in the cloud. The proposed model is suitable for implementing the blockchain structure, and the algorithm used for implementing the system model is efficient and can provide high security for data stored in the cloud. However, the latency, which is a limitation to be solved in this study, was not considered in their technique. In Srikanth, Somarouthu, et al. [26], the data to be stored by the user is encrypted, distributed, and stored in chunks. However, there is a difference from this study in that there is no verification or consensus on the data storage and download process. In Nandini and Girisha [27], their solution processes transactions with encrypted data based on smart contracts and is based on the Ethereum network, but the contents of large data storage are not considered. Huang, Pei, et al. [28] proposed a public third-party audit method that detects tampering on the cloud server to ensure reliability between the user who stores data and the cloud service. In this framework, all consensus nodes replace the third-party auditor to execute auditing delegations and record them permanently, thereby preventing entities from deceiving each other. To apply this method, however, it is necessary to interact with the cloud service provider through the framework and disclose the data to public nodes. This creates problems such as increased traffic on the central server and relying on a single point of failure. In this study, data are managed and verified in a single cloud, a cluster of blockchain nodes rather than validators. Li et al. [29] proposed a technique for storing and managing large-capacity data generated by IoT devices based on blockchain. In their study, data generated by IoT devices was stored in a distributed hash table (DHT), and the pointer address of the DHT was recorded in the blockchain. Our proposed architecture stores data in the local storage of each blockchain node rather than in an external DHT. Wang et al. [30] stored metadata through IPFS, a distributed file system, and the Ethereum blockchain to ensure the privacy and stability of data stored in cloud storage. In addition, data privacy was ensured through the ABE (attribute-based encryption) access control method. However, this method also distributed data and stored the data in a network outside the blockchain. ChainFS [31] proposed a middleware to ensure stability when end-users store data in cloud storage. Data were stored on the Amazon cloud, and secret-key distribution and file operations were recorded through the blockchain. In this paper, each node stored data and created, synchronized, and recovered service clusters. Segment blockchain  [32] aimed to reduce the storage requirements of the blockchain. To this end, each node in the blockchain uses a technique that stores only segments of the blockchain. In contrast to this, our proposed architecture reduces the storage requirements of nodes by storing user data in units of services and recording only metadata in the blockchain. The related works mentioned above are summarised in Table 1.

## 3. Architecture

### 3.1. System Architecture

**Figure 2:**
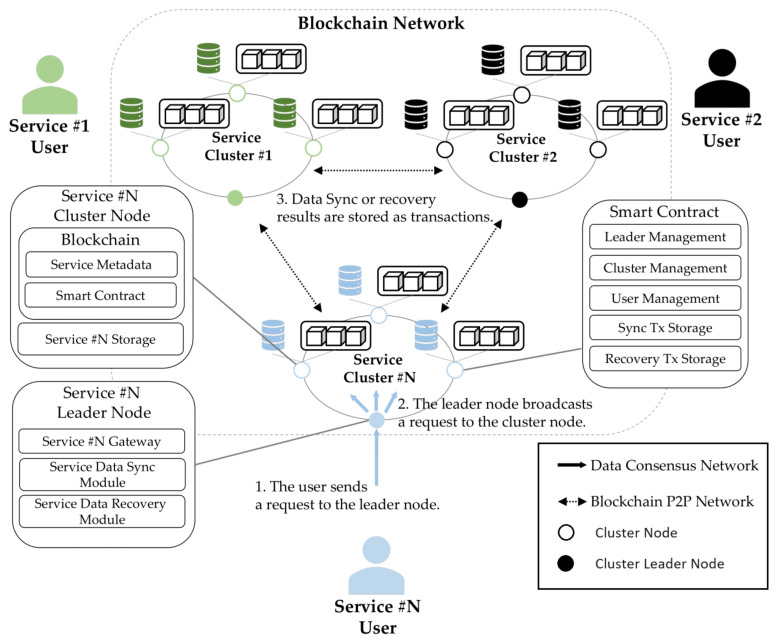
System Architecture.

Figure 2 shows the proposed architecture. In this architecture, two kinds of networks are implemented for service offerings. Firstly, the blockchain P2P network is responsible for blockchain maintenance, transactions, and block propagation within and between clusters. In addition, it deploys smart contracts to ensure reliability and synchronization of service data storage, service synchronization, and recovery transactions that can be stored and managed. In addition, it supports the management of the list of services by storing the service leader node that serves as a gateway for service creation, the cluster within the service, and the blockchain account and IP address of the cluster that stores data. Secondly, a configured data consensus network delivers file binary data (to be stored by users) through this data consensus network; metadata are generated and then propagated to the leader node. The consensus algorithm can use PoW, PoS, PBFT, etc. when used in a service cluster, and in this paper, it is composed of PoA-based Geth. In the case of data consensus algorithms, CFT and BFT are important in the process of verifying that the proof data are the same, so we use a PBFT-based consensus algorithm. The operation process is as follows:

- The service user sends a request for a data query or insert to the service leader node.
- In the service cluster, data are synchronized or recovered through the data consensus network within the service cluster.
- When the processing of data consensus in the cluster is finished, the leader node creates a transaction and records metadata in the blockchain.

### 3.2. Replacement Policy

**Figure 3:**
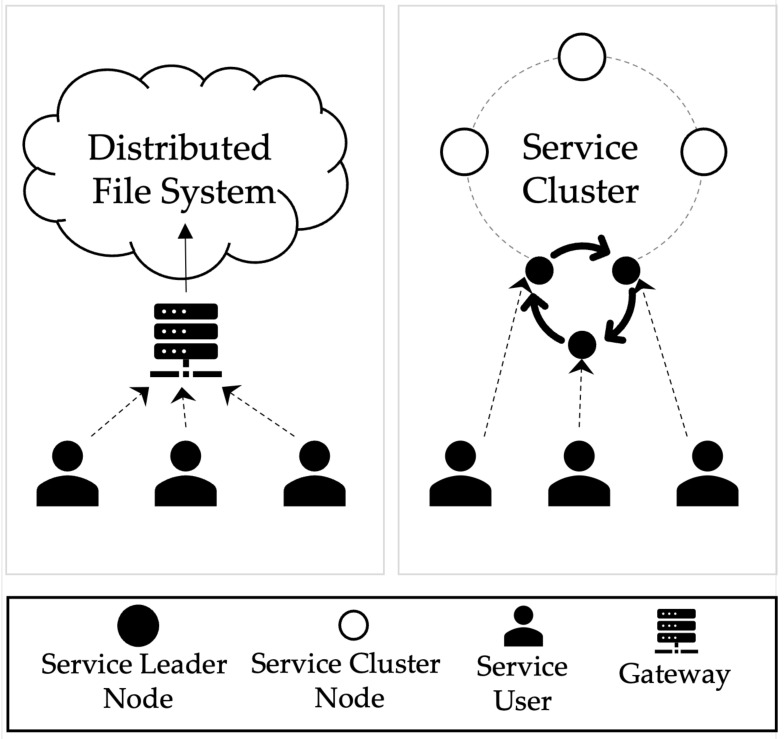
Method to replace the leader node for gateway distributed processing.

Figure 3 shows a proposed technique to solve the centralization of the gateway, a limitation of the existing distributed file system. Users initiate data storage and download requests to the central gateway to store data in the distributed file system. This method transmits data to multiple nodes, but the network traffic of the gateway increases, and the reliability of the data cannot be guaranteed. There are several methods that can be introduced to address these limitations: stake-based, randomized, and round-robin-based selection. In this paper, we implement the round-robin method of leader selection. The round-robin scheme we are talking about in this paper is a simple form of round-robin, where the next node acts as a gateway for each request. In this way, the service leader nodes, which act as gateways for the selected service clusters, can distribute traffic by sending and processing requests every specific round. This leader node is a normal node, such as a node acting as a gateway to another system, and typically takes no additional load beyond the load of maintaining the node. In addition, the proposed architecture records the metadata generated after the request of the cluster node in the blockchain, and consequently, the data transmitted to the gateway cannot be forged or falsified by verifying the signature.

In the proposed architecture, nodes in a blockchain network constitute a cluster of service units. A node consists of a service leader node for connecting users and services and a cluster node that stores data in local storage. A user must send a request to store data in a cluster of service units. However, one node is required to send all data to multiple cluster nodes and to aggregate data again for consensus. In addition, in a single node, traffic increases due to the overloading of the communication volume with the cluster node, and if a single node handles all communication, reliability problems occur during the transmission/reception process. Therefore, by applying the Round-Robin [33] method, the user’s request is propagated to all cluster nodes in the service, and the process for collecting responses is distributed and processed by the service leader nodes.

The proposed method solves the traffic increase problem of the gateway used in the existing distributed file system and guarantees reliability through the private key-based signature of the data delivered to the gateway and the data consensus process of the cluster nodes.

In addition, by applying this technique, our system can respond to cases where nodes are dynamically added and deleted in the public blockchain network environment. For example, if a node participating as a service leader node shuts down due to a crash fault, service processing in the cluster that the service leader node participated in may be interrupted for that round. In this case, the next service leader node in the queue is selected using the round-robin method, which allows recovery.

**Figure 4:**
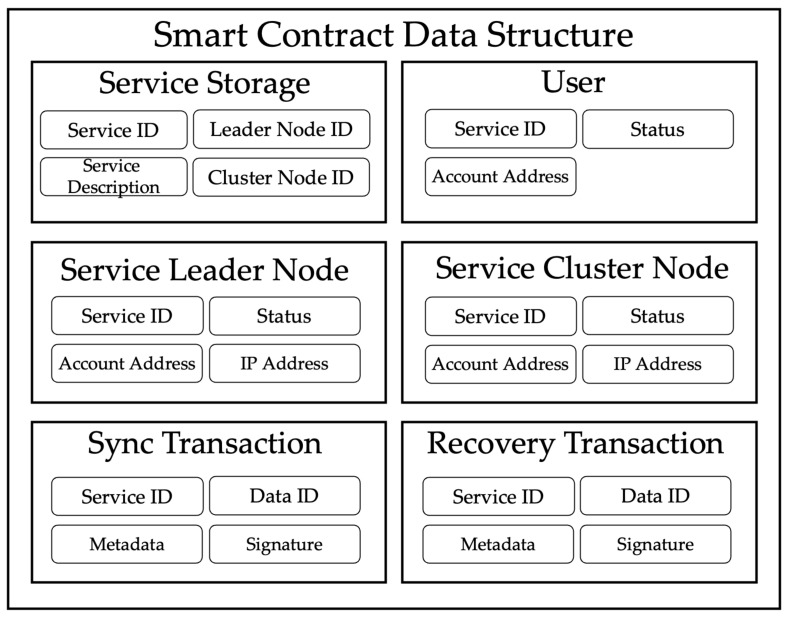
Data architecture of smart contract.

Figure 4 shows the data architecture of the smart contract for creating services, registering service users, registering leaders and cluster nodes, and storing synchronization and recovery transactions. The proposed architecture provides local storage for the nodes of the blockchain network rather than external storage by configuring a service cluster with nodes. This process is based on smart contracts executed by all blockchain network nodes, not by a single node. To do this, service creation, leader nodes, and synchronization are required, and all processes are performed through smart contracts.

### 3.3. Workflow

This section describes the process for saving data to the storage of blockchain nodes. The description of the term is as Table 2.

Algorithm 1 [Service creation and user and node registration process] is the process of configuring a service cluster in the blockchain network and registering the leader node of the service cluster, Cluster nodes that provide storage services to persist data, and User.

To configure the Network of Service Cluster for service provision as the node constituting the Network of Ethereum Blockchain Network, the service creator must request service creation from the smart contract. The service creator is created by registering as the leader node of the service cluster first. The created service is stored in a smart contract and can be checked by all nodes. When a registration request is made with a leader node, it is registered with the permission of the existing leader node of the service cluster.

In addition, cluster nodes that provide storage services to persist data of the service provider role that operates to maintain service data can register with a specific service through $ID_{service}$ of the service and blockchain account and IP.

Service User can check the list of service clusters created in the blockchain network. User can register for the service and request data storage and retrieval through a transaction that includes a blockchain account.

Algorithm 2 [Process of synchronizing the data of the data store and cluster nodes] is the data storage process. The process of this algorithm is shown in Algorithm 2; User requests IP of the leader node of the service to store Data in the network of service clusters and the leader node of the service cluster Send Request Type of STORE. After the data storage process is completed, the user returns $ID_{data}$. The leader node of the service cluster that receives Request verifies the account of User and checks whether it is registered in the service. If verified, Request is sent to all cluster nodes that provide storage services registered in the service. The network of service cluster nodes receives Request and stores it in $DB_{cluster}$. The network of service clusters encrypts the hash of the saved file with $Key_{priv}$ to create Sig, creates Metadata, including FileHash and Sig, and sends it to the leader node of the service cluster. The leader node counts whether all cluster nodes in the network of service clusters have saved data and sends Metadata of all cluster nodes to the smart contract to $Tx_{sync}$ is created. In a smart contract, the data of Metadata are verified with the Keypub of cluster nodes, and the same FileHash is created. When $Tx_{sync}$ is saved, FileHash is broadcast to all cluster nodes so that the saved FileHash can be verified.
**Algorithm 1** The process of crafting service**Procedure:** THE PROCESS OF REQUEST SERVICE AND ENROLLMENT NODE FROM SC 1:ServiceCreatorInfo←Account,IP,Status 2:$ID_{service},ID_{leader}\leftarrow$CreateNewService(ServiceCreatorInfo) 3: **Procedure:** THE PROCESS OF CREATING SERVICE FROM SC 4:$ID_{service}=Count\left(ServiceList\right)+1$ 5:$ServiceIndex\leftarrow ID_{service},ServiceType$ 6:NodeList←LeaderList,ClusterList 7:TxList←SyncTxList,RestoreTxList 8:ServiceInfo←ServiceIndex,NodeList,TxList 9:ServiceList.Add(ServiceInfo)10: **Procedure:** THE PROCESS OF ADDING $Node_{leader}:$11: 12:**if** $CheckServiceLeaderList(ID_{service},Account,IP)$ is not **then**13:    $LeaderInfo\leftarrow LeaderInfo(ID_{service},Account)$14:    $ID_{cluster}\leftarrow AddLeader\left(LeaderInfo\right)$15:**end if**16: **Procedure:** THE PROCESS OF ADDING $Node_{cluster}:$17: 18:**if** $CheckServiceClusterList(ID_{service},Account,IP)$ is not **then**19:    $ClusterInfo\leftarrow ClusterInfo(ID_{service},Account)$20:    $ID_{cluster}\leftarrow AddCluster\left(ClusterInfo\right)$21:**end if**22: **Procedure:** THE PROCESS OF ADDING User:23: 24:**if** $CheckServiceAccountList(ID_{service},Account)$ is not **then**25:    $UserInfo\leftarrow ServiceUserInfo(ID_{service},Account)$26:    $ID_{user}\leftarrow AddUser\left(UserInfo\right)$27:**end if**

**Algorithm 2** The Process of storing service data
**Procedure:** THE PROCESS OF Posting Request FROM User To $Node_{leader}$ 1:$IP_{leader}$← GetCurLeaderNode($ID_{service}$) 2:$ID_{data}$← PostRequest($ID_{service}$, $IP_{leader}$, $ID_{user}$, Type, Address, File) 3: **Procedure:** THE PROCESS OF Broadcasting Request FROM $Node_{leader}$ To $Node_{cluster}$ 4:$IpTable_{cluster}$← GetClusterNodeList($ID_{service}$) 5:Metadata = $BroadcastRequest(IpTable_{cluster},Request)$ 6:**if** Count(Metadata) is $Count(Network_{cluster})$ **then** 7:    $Tx_{sync}$$\leftarrow CreateSyncTx(Key_{Priv})$ 8:    $StoreSyncTx(Tx_{sync})$ 9:
**end if**
10: **Procedure:** THE PROCESS OF Storing Data FROM $Node_{cluster}$11:

Reqeust←GetRequest()

12:

PATH←StoreDataLocalStorage(Reqeust.File)

13:

FileHash←GenerateSig(Reqeust.File)

14:

$Sig\leftarrow GenerateSig(FileHash,Key_{priv})$

15:

Metadata←GenerateMetadata(Sig,FileHash)

16:

ResponseMetadata(Metadata)




**Figure 5:**
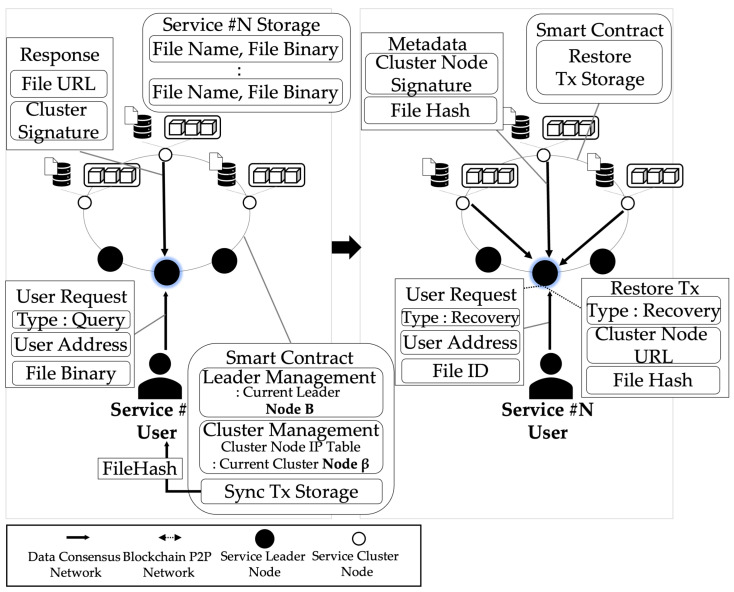
The process of query and validating service data.

Algorithm 3 [Process of downloading and verifying data] is the process of downloading and verifying data. The process of this algorithm is shown in Algorithm 3 and Figure 5. This shows the process of querying and verifying Data stored in the network of service clusters by User. User needs $ID_{data}$ of stored data and the IP of the current leader node of the service cluster to return Data. User sends Request to the leader node of the service cluster $ID_{data}$ and user’s blockchain Address and $ID_{service}$ via $IP_{leader}$. Leader nodes of the service cluster delivers Requests of query type to $CurNode_{cluster}$(current leader node of the service cluster) selected in a round-robin method. The current leader node of the service cluster creates a URL accessible through PATH stored in $DB_{cluster}$ with $ID_{data}$, and encrypts URL with $Key_{priv}$ to create Sig. current leader node of the service cluster creates Metadata through URLandSig and sends it to User through $Node_{leader}$. User downloads FileBinary through a URL and creates a FileHash. Additionally, User is compared with FileHash recorded in the network of the ethereum blockchain network to verify that the data are synchronized. If FileHash is different, the recovery process is performed.

Algorithm 4 [Process of data recovery] is the recovery process in case the verification is performed after data download. This process occurs when the data returned by User in Algorithm 3 is tampered with or out of sync. This process is performed to keep the data of all service cluster nodes the same through data consensus. Additionally, the client can return synchronized data after this process. If verification of Data fails, User requests data recovery from the leader node of the service cluster, and $Tx_{recovery}$ is saved in a network of the ethereum blockchain. User sends Request of Recovery Type to the leader node, and the current leader node broadcasts it to all cluster nodes that provide storage service in the network of service clusters. Cluster nodes that provide storage services sends the FileBinary of $ID_{data}$ i ncluded in Request to the leader node. When transmitting, every leader node of the service cluster divides FileBinary into N equal parts to create a FileHash. This process decentralizes the creation and verification of FileHash to be performed by multiple leader nodes in the service cluster. Additionally, the current leader node of service cluster sends a save request to the service contract to create $Tx_{restore}$ by collecting FileHashandURL created by the leader node of the service cluster. In a smart contract, the FileHash of $ID_{data}$ determines another cluster node as the generated FileHash and sends a URL for recovery to the cluster nodes.
**Algorithm 3** The process of querying and validating service data**Procedure:** THE PROCESS OF CREATING QUERY Request FROM User 1:$IP_{leader}$← GetCurLeaderNode($ID_{service}$) 2:URL,Signature← PostRequest($ID_{service}$, $IP_{leader}$, $ID_{user}$, Type, Address, $ID_{data}$) 3: **Procedure:** THE PROCESS OF POSTING Request FROM $Node_{leader}$ 4:$IP_{cluster}$← GetCurClusterNode($ID_{service}$) 5:$URL,Signature\leftarrow BroadcastRequest(IP_{cluster},Request)$ 6: **Procedure:** THE PROCESS OF BROADCASTING URL FROM $Node_{cluster}$ 7:$PATH\leftarrow ReadDataLocalStorage(Reqeust.ID_{data})$ 8:URL←GenerateURL(PATH) 9:$Sig\leftarrow GenerateSig(URL,Key_{priv})$10:Metadata←GenerateMetadata(URL,Sig)11:ResponseMetadata(Metadata)12: **Procedure:** THE PROCESS OF VERIFICATING Data FROM User13:$FileHash\leftarrow GetFileHash(ID_{data})$14:File←GetFileBinary(URL)15: 16:**if** GetFileHash(File) is not FileHash **then**17:    $IP_{leader}$← GetCurLeaderNode($ID_{service}$)18:    URL,Signature← PostRequest($ID_{service}$, $IP_{leader}$, $ID_{user}$, Type, Address, $ID_{data}$)19:**end if**

**Algorithm 4** The process of recovering service data
**Procedure:** THE PROCESS OF BROADCASTING $Tx_{recovery}$ FROM $Node_{leader}$ 1:

$BroadcastRequest(IP_{cluster},Request)$

 2:  3:**if** $Node_{leader}$ is $CurNode_{leader}$ **then** 4:    FileHash,Sig←GetRecoveryResponse() 5:    FileHashes,Signature← PostRequest($ID_{service}$,$IP_{leader}$,$ID_{user}$,Type,Address, $ID_{data}$) 6:    **if** Count(FileHashes) is $Count(Network_{leader})$ **then** 7:        $Tx_{restore}$$\leftarrow CreateRestoreTx(Key_{Priv},FileHashes)$ 8:        $StoreRestoreTx(Tx_{restore})$ 9:    **end if**10:**else if** $Node_{leader}$ is not $CurNode_{leader}$ **then**11:    URL,Sig←GetRecoveryURL()12:    File←GetFileBinary(URL)13:    Result←VerifyFile(GetFileHash(File),FileHash)14:    PostVerificationResult(Result)15:
**end if**



## 4. Experiment

### 4.1. Experiment Setup

In this section, we describe the results of experiments conducted to measure and compare the performance of the proposed architecture. For the experiment, User, cluster nodes that provide storage services, and leader node of the service cluster were configured on physically separated devices. The experimental environment in Table 3 is as follows. User sent a request to the leader node via gRPC communication based on WiFi (802.11ac) in a Laptop with an Apple M1 Chip and 16 GB of RAM. The leader node runs on the Dell EMC PowerEdge R740 server (CPU: Intel® Xeon® Silver 4210R 2.4 G, RAM: 32 GB RDIMM), and a total of 3 nodes are configured as Docker containers locally on the server. The Cluster node is a desktop (Gen Intel(R) Core(TM) i9-11900KF, RAM 32.0 GB), and 3 nodes are configured as a Docker container unit. In the experiment, User, cluster node, and leader node were included in different networks, so that the cluster node and the leader node were each assigned an IP. Since the goal of this experiment was to see how well we could distribute traffic rather than address scalability, we configured the network with a total of three nodes. The experiment measured and compared file data storage and query latency from the client’s point of view and measured data storage and recovery agreement and data storage latency during the operation of the proposed architecture.

### 4.2. Experiment Data

The performance evaluation criteria compared latency with IPFS, which is the most widely used off-chain. To measure the latency and performance of service data storage, the data size of the currently most used file format was organized as shown in Table 4.

### 4.3. The Latency Service Data I/O

**Figure 6:**
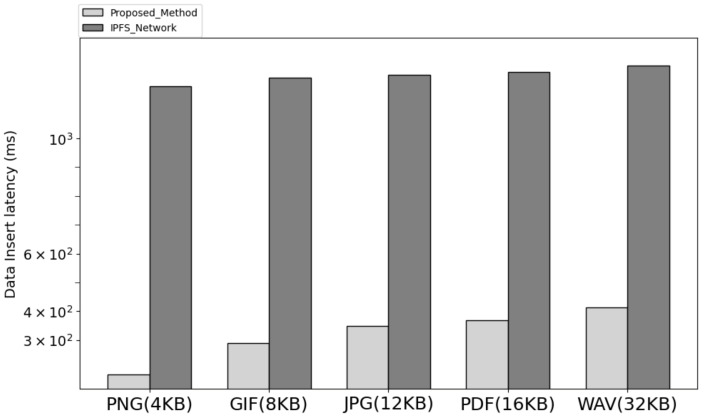
The latency insert compare with proposed method and IPFS.

Figure 6 is the result of comparing the data input time of the network implementing the proposed system architecture and IPFS. In the proposed architecture, the user sends a request including the file binary and account information to the service leader node, and the service leader node broadcasts the request to the cluster node. The cluster node delivers the requested metadata to the service leader node, and the leader node combines them and transmits them to the smart contract. In the smart contract, the synchronization transaction is stored through the data transmitted by the service leader node, and the data of all cluster nodes is confirmed to be synchronized. In the process, when the latency from the time the user transmits the request to the synchronization transaction request to the smart contract was measured, the latency was lower than when the data of the same size were stored in the IPFS.

**Figure 7:**
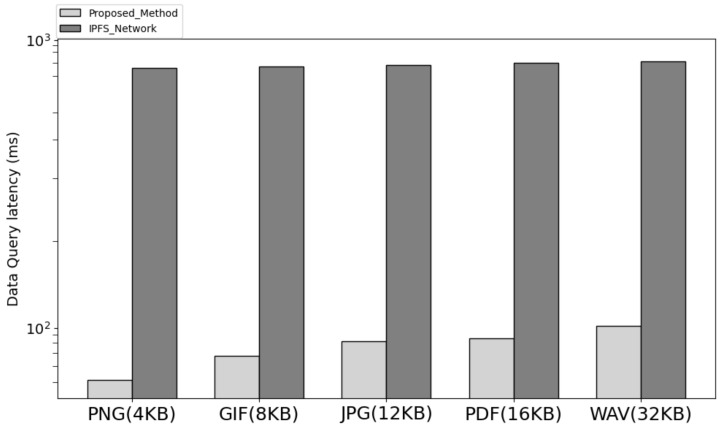
The latency query compare with proposed method and IPFS.

Figure 7 is the result of comparing the data download request processing time of the network implementing the proposed system architecture and IPFS. In the proposed architecture, the user sends a request, including the ID of the returned data, to the service leader node after saving the data to request data. The service leader node transmits a request to the node corresponding to the current cluster node in a round-robin manner among cluster nodes. Upon receiving the request, the cluster node verifies the user’s address and signature and sends the data-accessible URL and URL to the user through the service leader node, including the encrypted signature with the cluster node’s private key. Users can download data through the URL. The latency in the process was further reduced by using the method of downloading binaries from a single node compared with the IPFS method, in which files are distributed and stored by multiple nodes. Therefore, it showed lower latency compared with the method of uploading data from IPFS and downloading distributed files through CID and showed a larger difference compared with the difference in data storage time.

### 4.4. The Latency of Processing Synchronization

**Figure 8:**
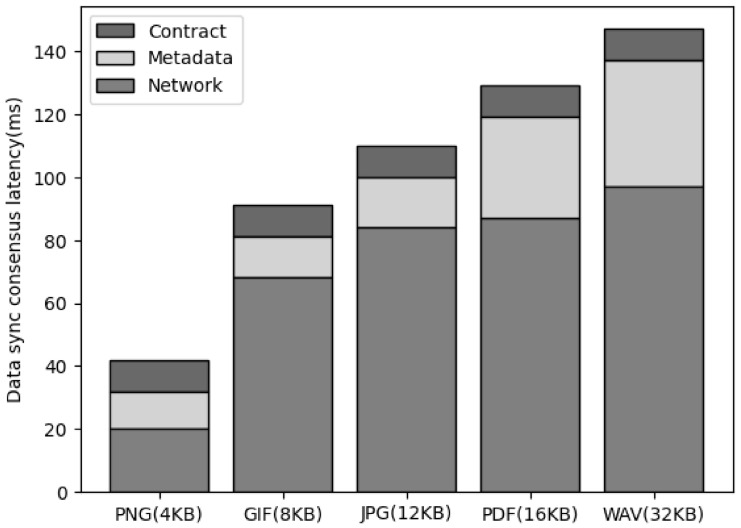
The comparison of the sync consensus latency of data size.

Figure 8 is the result of measuring the latency for data consensus in progress to ensure synchronization of all cluster nodes for data when saving data in the proposed system architecture. To store the synchronization transaction, the file binary data are passed, and the cluster node must store the binary data and generate metadata, including the file hash and signature. In addition, the service leader node sends a request to store the synchronization transaction in the smart contract through the corresponding metadata. This process is included in the process of data consensus by storing the synchronization transaction. As a result of measuring this process, there was no significant difference according to the data size, and there was no large latency in the smart contract call or metadata generation latency. In the case of network latency, it increased as the data size increased, but it was confirmed that the increase did not significantly affect the performance.

### 4.5. The Latency of Processing Recovery

**Figure 9:**
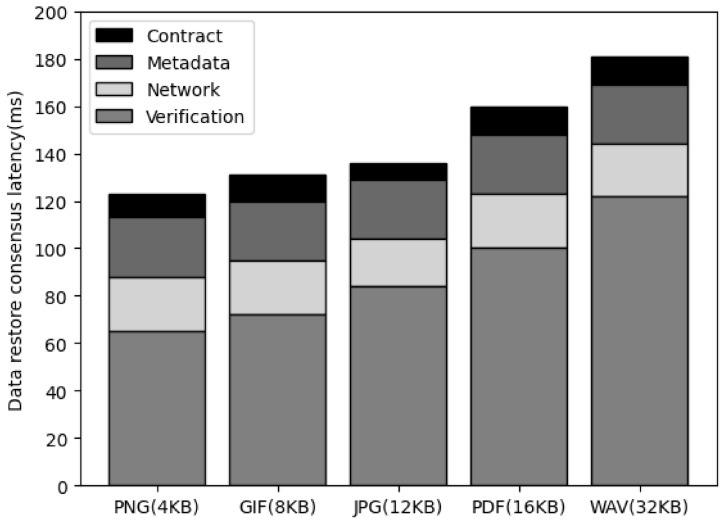
The comparison of the restore consensus latency of data size. (When replacing service leader node from 1 to 2).

Figure 9 shows the latency for the data recovery consensus process by sending a request to the service leader node when the user verifies the data after downloading the file and if the verification fails. When a user sends a request to the service leader node, the leader node forwards the request to all cluster nodes. The cluster node forwards the data included in the request to all leader nodes. All leader nodes generate a FileHash value from file data, encrypt it with their private key, and transmit it to the current leader node, and the leader node calls a smart contract to save the recovery transaction. When the transaction is saved and the block is propagated, the cluster node checks whether the data has been restored through a smart contract event and requests the data through the URL to synchronize the data. In this process, the latency includes the process of requesting the user to download a file from the cluster node, verifying the file hash value through the data recorded in the blockchain, and delivering it to the cluster node through the service leader node. It also includes the process of sending the file binary data from the cluster node to the leader node and generating metadata, as well as the smart contract request time when the leader node currently selected as a round robin sends a recovery transaction storage request. As a result of the measurement, the latency for downloading the file binary data and verifying it by the user was the largest, and the latency for contract calls and metadata creation was constant. In the case of file transfer time, the latency increased according to the file size, but the increase was not large.

### 4.6. The Latency of Service Recovery Due to the Crash Fault of Service Leader Node

**Figure 10:**
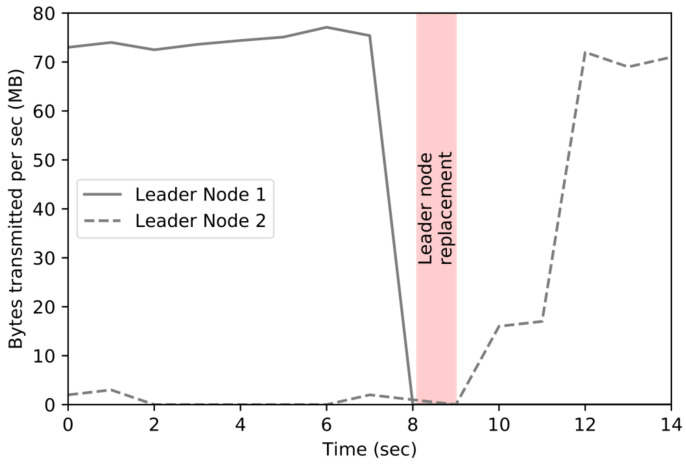
The latency leader node replacement.

Figure 10 shows the result of a comparison with the architecture in which the proposed leader replacement policy is applied to solve the traffic increase problem of the centralized gateway of the existing distributed file system. For the experiment, the user’s request was transmitted at the same interval when the leader node replacement method was applied and when it was not applied. In the case of leader node replacement, the three leader nodes change their order every 0.9 s in a round-robin manner and receive user requests. As a result of the experiment, when the gateway was centralized, the overall traffic increase was high for both data delivery and transmission from the leader node. When the user’s request data are transmitted, network traffic is about three times higher when there is one leader node than when three nodes are changed in a round-robin method. When user data are transmitted to the cluster node, the amount of traffic generated is higher than that of input traffic, and it is twice as high as when round-robin is applied.

### 4.7. The Latency Analysis of Cluster Node Storage Processing

**Figure 11:**
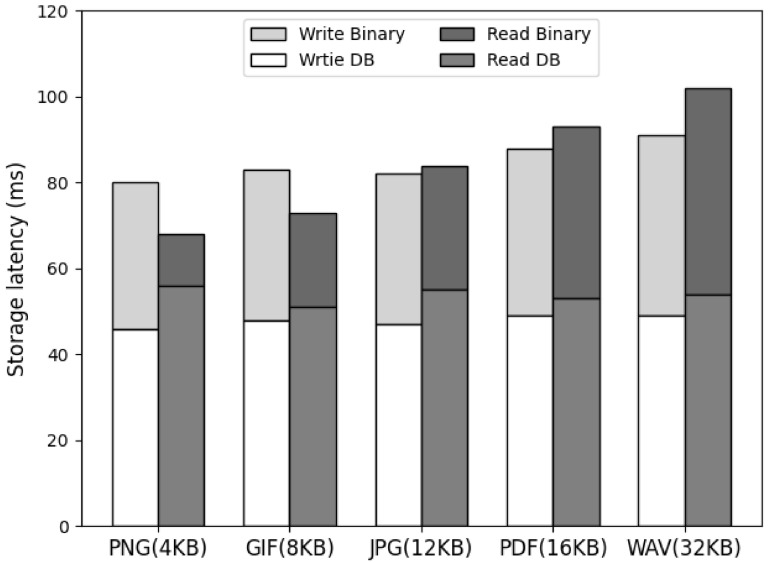
The comparison storage processing latency.

Figure 11 shows the result of measuring the latency when the cluster node stores and queries the file binary data. In this paper, we aim to reduce the latency that occurs when storing and querying file data using the file data management technique based on the existing external network. Both the network speed and file-data storage latency of the cluster node storing the data must be minimized. To this end, the file index was managed based on NoSQL, and the file data was stored in the local storage of the cluster node. Our results show no significant difference between the data storage path indexing latency and the file data storage latency for the data save request, and the read request had a higher latency for reading the file binary data. Both operations showed small latency that did not affect performance, and the increase in latency was not large depending on the file size.

## 5. Conclusions

In this paper, we propose a blockchain architecture to improve network latency and reliability, which is a limitation of technologies that use external off-chain networks due to the inability to store large amounts of data as the size of the blockchain increases when storing data generated by IoT devices. To reduce latency, blockchain nodes are clustered as a service unit to store and query data in local storage. The reliability of the data was ensured. When compared against existing off-chain alternatives, the results of our proposed architecture showed lower latency, and the local storage data processing speed of the cluster node and the latency in each process had a significant impact on performance. The experiments confirmed that there was no detrimental effect on performance. Additionally, unlike the existing data storage methods such as cloud storage and distributed file systems, the nodes of the blockchain network duplicate the data source, and the client directly verifies the tampering of the data and requests the data of the cluster nodes to synchronize the data to the original data. This guarantees reliability and reduces the possibility of data loss. In subsequent studies, distributed storage techniques are applied to solve the limitation of the local storage increase in each cluster node that is configured in service cluster units and maintains service binary file data in the blockchain network, an efficient replacement policy, and the signatures of leader nodes and cluster nodes. We plan to study how to reduce the gas consumption of smart contracts used for verification. In addition, we will refer to the comparative analysis of consensus algorithms [34] to identify the characteristics of each consensus algorithm and use the identified characteristics to make further improvements to the consensus algorithms we performed or supported in this study. Plus, as the availability of IoT services expands, the system proposed in this paper can be widely used in official document processing services and patent registration [35].

## Figures and Tables

**Table 1:** Comparison of the existing blockchain storage.

	Abstract	Contribution	Differences from the Proposed Structure	Organize
Web3Storage [19]	- Store data in IPFS of 3 geographically distributed nodes hosted by Protocol Labs. - Data will then be stored in at least 5 decentralized miners on the Filecoin network.	- Storing data on an IPFS network in a decentralized network, providing an easy interface through hosting - Self-hosted data exists.	- It hosts a separate node directly and stores the data, so the stability of the stored data are not taken into account. - There is a delay in the process of data transmission to users, IPFS Cluster, and Filecoin network.	o Data Stability − o High Latency − o Easy Interface +
STORJ [20]	- Store metadata on the Ethereum blockchain. - Record data by sharding and distributing it.	- Metadata are created with information about where the data can be found again. - The original data are merged back into the client’s local system. - Data tampering is verified through regular inspection of data through parity shards.	- There is a delay in the process of encrypting, dividing, saving, and downloading files. - Storj bridges exist, so single point errors are possible. - Encryption, partitioning, and combining of data are performed locally on the client, so there is a load on the client side.	o High Latency − o SPOF possibility − o Load on Client − o Validate Data Tampering +
BigChainDB [21]	- Each node maintains a locally independent mongo DB, stores data, and proceeds with consensus based on Tendermint.	- BigchainDB uses Tendermint to cope with the BFT problem by making the entire network work properly even if up to a third of the nodes fail. - Because the subject of the network manage the list of nodes, a malicious person or organization cannot attack the network using a large number of nodes. - To request processing of BigchainDB’s network, BigchainDB HTTP API is used.	- Since the network subject exists and manages the node list, the reliability of the node cannot be guaranteed. - Since clusters cannot be configured in units of services, a large files cannot be stored.	o Data Reliability − o Cannot Store Large File − o Hard to Attack + o Fault Tolerance +
BlockHouse [24]	The methodology of Blockhouses focuses on a method that contains three components: initialization of the storage device, day-audits, and conclusion of the device.	- Except the data transferred between the client and the server, all the actions go through a smart contract in the blockchain in order to log, pay and secure the entire storage process. - This system uses a dual Smart Contract and Proof of Retrievability system to automatically check at a fixed frequency if the file is still hosted.	- The degree of decentralization is low by using a private blockchain. The main problem that happens in the network may be that the scale of the blockchain is too drastically it is impossible to store. By canceling production the erasure codes are used to rectify the problem.	o Decentralized −
In Pise, et al. [25]	The system uses proof of storage and proof of work to verify that hosts do not meddle with data in blockchain.	- It uses Space Wallet, a special structure that tracks available storage space on all nodes - The proposed system does not encrypt or decrypts data before uploading it to peers which creates a threat to confidentiality and privacy of user’s data.	- it does not solve the file recovery issue at the end of the storage. - It does not encrypt data and requires CPU computation by using PoW method.	o Cannot Recover Data − o Load local CPU −
Srikanth et al. [26]	Using your PC as a storage server by storing data on unused storage space.	- the user’s file is encrypted and stored across multiple peers in the network using the IPFS(InterPlanetary File System) protocol.	- By using IPFS as an off-chain file, only the hash value is recorded in the blockchain after encrypting and distributed storage. - It did not solve the data reliability and latency problem of IPFS.	o Data Reliability − o High Latency −
In Nandini et al. [27]	For efficient storage use, data are stored in a storage space not used by the existing PC, using the PC as a storage server, and focusing on data security.	- Hashing Algorithm-It is created using a hashing function such as 256(SHA 256) and stores the hash value of encrypted chunks. - At the client end, a hashing function such as SHA 256 is used to store chunked data.	- Data to be stored on the user side is encrypted, divided into chunks and stored in distributed nodes. - In the storage process, there is no consensus of nodes and no stability and reliability verification process. - There is no verification process for data when downloading data.	o Data Stability − o Data Reliability −

**Table 2:** Notation for Algorithm Representation.

Symbols	Description
$ID_{service}$	Index that identifies the created service
$ID_{leader}$	Index that identifies the Leader Node
$ID_{cluster}$	Index that identifies the Cluster Node
$ID_{data}$	Index that identifies the service Data
$IP_{leader}$	Internet Protocol address of the leader node
$IP_{cluster}$	Internet Protocol address of the cluster node
$Network_{bc}$	Network of Ethereum Blockchain Network
$Network_{cluster}$	Network of Service Cluster for service provide
$Node_{bc}$	Blockchain Network Node
$Node_{leader}$	Leader node of the service cluster acting as a gateway
$Node_{cluster}$	Cluster nodes that provide storage services to persist data
User	User of blockchain storage service
$CurNode_{leader}$	Leader node of the service cluster acting as a gateway
$CurNode_{cluster}$	Cluster nodes that provide storage services to persist data
$DB_{cluster}$	Database for service cluster data management
PATH	The path of data stored
SC	Smart Contract deployed on $Network_{bc}$
Request	User requests to store and query data
$Tx_{sync}$	Transaction for data sync
$Tx_{recovery}$	Transaction for data recovery
$Data_{service}$	Service Data (MP3, HTML, JPEG)
Metadata	Metadata for data verification
$URL_{data}$	URL for service data access
Account	Blockchain Account
$Key_{pub}$	Public Key
$Key_{priv}$	Private Key
Sig	Signature generated by encryption with the private key to verify the result

**Table 3:** Experiment Enviroment.

Type	Name	Function	Specs (Version)
HW	Server	Running Service Leader Node	DellEMC Power Edge R740 server (CPU: Intel Xeon Sliver 4210R 2.4 G, RAM: 32 GB RDIMM, Ubuntu 18.04)
	Desktop	Running Service Cluster Node	Gen Intel(R) Core(TM) i9-11900KF, RAM 32.0 GB, Window 10
	Laptop	Running User	Apple M1 Chip, RAM 16 GM
SW	Docker	Node operation	version 20.150.7
	Golang	Used to implement node	version 1.18
	MongoDB	Database on cluster nodes	version 6.0
	Redis	Pub-sub	version 7.0
	Solidity	Smart contract	0.8.7

**Table 4:** Dataset.

Type	PNG	GIF	JPG	PDF	WAV
**Size**	4 KB	8 KB	12 KB	16 KB	32 KB

## Data Availability

Not applicable.
